# OP44 Cost-Of-Illness And Cost–Consequence Analysis Of Dementia In Italy

**DOI:** 10.1017/S0266462324001065

**Published:** 2025-01-07

**Authors:** Francesco Saverio Mennini, Chiara Bini, Paolo Sciattella, Nicola Vanacore

## Abstract

**Introduction:**

In Italy, there is a lack of evidence regarding the care and management of patients with dementia, as well as the associated costs. This study aims to fill this informational gap by utilizing data from both literature and national surveys.

**Methods:**

A prevalence-based cost-of-illness (COI) model was developed to assess dementia-related costs from a societal perspective. The resources utilization for management and treatment of patients with dementia was derived from both the literature and the analysis of surveys conducted by the National Institute of Health on the social-health structures dedicated to dementia. Indirect costs from informal caregiving were evaluated through a human capital approach. Additionally, a cost–consequence analysis (CCA) was conducted to assess the economic impact of healthcare resource utilization changes.

**Results:**

Based on an estimated 1,150,691 dementia cases in Italy, with approximately 12 percent institutionalized, the COI model estimated an annual expenditure of around EUR23.6 billion (USD25.7 billion) for dementia patient management, with 63 percent attributed to out-of-pocket expenses. CCA indicated that if all affiliated with Centers for Cognitive Disorders and Dementia (CDCD) received nonpharmacological interventions (versus the surveyed 25.5 percent), there would be a direct cost increase of approximately EUR4.3 million (USD4.7 million).

**Conclusions:**

This analysis provides an updated overview of current dementia patient management in Italy, offering valuable insights for decision-makers to prioritize health policies and interventions for patients and their caregivers.

